# Increased Serum Levels of IFN-*γ*, IL-1*β*, and IL-6 in Patients with Alopecia Areata and Nonsegmental Vitiligo

**DOI:** 10.1155/2020/5693572

**Published:** 2020-08-03

**Authors:** Katarzyna Tomaszewska, Magdalena Kozłowska, Andrzej Kaszuba, Aleksandra Lesiak, Joanna Narbutt, Anna Zalewska-Janowska

**Affiliations:** ^1^Psychodermatology Department, Clinical Immunology and Rheumatology, Medical University of Lodz, Pomorska 251, 92-213 Lodz, Poland; ^2^Department of Dermatology, Pediatric Dermatology and Oncology, Medical University of Lodz, Kniaziewicza 1/5, 91-347 Lodz, Poland

## Abstract

Alopecia areata (AA) and vitiligo are both common skin diseases of autoimmune origin. Both alopecia areata and vitiligo have shown to be affected by oxidative stress. The present work is aimed at evaluating and comparing the serum proinflammatory cytokine levels in AA and nonsegmental vitiligo (NSV). A cross-sectional study was conducted of 33 patients with AA, 30 patients with NSV, and 30 healthy controls. Serum levels of interferon *γ* (IFN-*γ*), interleukin- (IL-) 1*β*, and IL-6 were determined quantitatively by ELISA method. Our analysis identified a *signature* of *oxidative stress* associated with AA and NSV, characterized by elevated levels of IFN-*γ* (AA: *p* = 0.007283; NSV: *p* = 0.038467), IL-1*β* (AA; NSV: *p* ≤ 0.001), and IL-6 (AA; NSV: *p* ≤ 0.001). IL-6 was also significantly increased in NSV patients in comparison with AA patients (*p* = 0.004485). Our results supported the hypothesis that oxidative stress may play a significant role in promoting and amplifying the inflammatory process both in AA and vitiligo. The complex understanding of both disease etiopathogenesis involves interrelationships between oxidative stress and autoimmunity. The clinical study registration number is RNN/266/16/KE.

## 1. Introduction

Alopecia areata (AA) and vitiligo are both autoimmune diseases, and striking similarities in pathogenesis have been identified at the level of both the innate and adaptive immune systems. Increased reactive oxygen species and high cellular stress levels have been suggested as the initiating trigger of the innate immune system in both diseases, and genome-wide association studies have implicated risk alleles that influence both innate and adaptive immunity [1–3]. Both conditions are known to carry a considerable impact on health-related quality of life [4, 5].

AA is a nonscarring hair loss with an unpredictable course and a wide spectrum of manifestations. It affects both genders equally with a cumulative lifetime incidence of about two percent and no significant racial predominance [6]. The most frequent clinical presentation of AA is in single or multiple patches. Occasionally, AA may progress to complete baldness, which is referred to as alopecia (areata) totalis (AT). When the entire body suffers from complete hair loss, it is referred to as alopecia (areata) universalis (AU). Ophiasis is a form of AA characterized by the loss of hair in the shape of a wave at the circumference of the head [7].

Vitiligo is an acquired disorder of the skin and mucous membranes that is characterized by well-circumscribed, depigmented macules and patches that occur secondary to selective destruction of melanocytes [8]. Vitiligo itself has been classified based on clinical grounds into two major forms, namely, segmental vitiligo (SV) and nonsegmental vitiligo (NSV), the latter including several variants (generalized vitiligo, acrofacial vitiligo, and universal vitiligo) [9]. NSV is the most common form of the disease (accounting for 85 to 90% of cases overall) and is associated with an increased risk of autoimmune diseases, especially Hashimoto's thyroiditis [10].

Due to limited knowledge regarding the role of systemic cytokine profiles associated with oxidative stress in AA and vitiligo, we aimed to determine and compare the serum levels of interferon *γ* (IFN-*γ*), interleukin- (IL-) 1*β*, and IL-6 in AA and NSV patients. Whether the pattern 7of serum cytokines could be associated with clinical details and disease activity in patients was also investigated. Previous studies had shown that both Th1 and Th17 cells are involved in the development of AA and NSV. Proinflammatory cytokines of innate immunity such as IL-1*β* and IL-6 with transforming growth factor *β* (TGF-*β*) are essential for Th1 and Th17 differentiation [11, 12]. IFN- *γ* is crucial for potentiating the activity of CD8+ T cells and natural killer cells in AA and NSV [13]. IL-15 stimulates the proliferation and activation of T cells, macrophages, CD5 memory lymphocytes, and cytotoxic CD8 lymphocytes [14]. IFN-*γ*, IL-1*β*, and IL-6 are also known as oxidative stress triggers.

## 2. Materials and Methods

### 2.1. Materials/Study Subjects

The study included 33 patients with AA and 30 patients with NSV. The control group consisted of 30 healthy individuals. All patients were of caucasian ethnicity. The diagnosis of AA and NSV was based on a detailed medical history and clinical and dermoscopic/trichoscopic examination. Clinically ambiguous cases, patients with a sign of infection, and patients with a history of using topical treatment within the past 2 weeks or systemic treatment within the past 4 weeks were excluded from the study. A detailed history and clinical examination were recorded for each patient. Controls consisted of healthy individuals of similar age who do not have alopecia areata or vitiligo and other comorbidities.

The group of patients with AA included 21 females (63.6%) and 12 males (36.4%), and the group of patients with vitiligo included 18 females (60%) and 12 males (40%). The control group contained 23 females (76.7%) and 7 males (23.3%) subjects. The mean ages of AA patients, NSV patients, and healthy controls were 18.64 ± 8.56 years, 28.55 ± 19.23 years, and 19.95 ± 13.08 years, respectively. There were no statistical differences in sex (c2 test) and age (*t*-test) between the patients and the normal controls.

To assess the clinical extent of AA and NSV, we calculated the Severity of Alopecia Tool (SALT) [15] and Vitiligo Area Severity Index (VASI) [16] scores, respectively. The activity of both AA and NSV was assessed at the Vitiligo Disease Activity (VIDA) Score [17].

The study was approved by the Bioethics Committee of the Medical University of Lodz (RNN/266/16/KE) and was conducted following the Declaration of Helsinki. All subjects gave informed consent to participate in the study.

### 2.2. Methods

Peripheral venous blood samples were collected from all patients and healthy controls. Sera were isolated by centrifugation and stored at –70°C before analysis and the concentrations of cytokines (pg/ml), namely, IFN-*γ*, IL-1*β* (Diaclone, Besancon Cedex, France), and IL-6 (Gen-Probe Inc., San Diego, California, USA) were determined quantitatively in collected serum samples by the enzyme-linked immunosorbent assay (ELISA) method in both patients and controls. ELISA tests were performed according to the manufacturer's instructions. The serum cytokine levels were compared between groups. The correlation of serum cytokine levels with sex, extent, activity, and duration of disease was studied.

### 2.3. Statistical Analysis

Statistical analysis was carried out with the Statistica software version 12 (StatSoft, Tulsa, OK, USA). To determine the distribution of quantitative variables, the Shapiro-Wilk test was used. The Mann–Whitney test was used to compare the median serum cytokine levels between the groups. Correlation analysis was determined using the Spearman rank correlation test. Data were considered to be statistically significant at a value of *p* < 0.05.

## 3. Results and Discussion

### 3.1. Results

#### 3.1.1. Demographic and Clinical Characteristics

The demographic and clinical characteristics of study participants are described in Table 1. Of the 33 enrolled patients with AA, 26 had patchy AA (SALT: S1 ≥ S4a) and 7 had severe disease (SALT: S4b ≥ S5). All of the 30 enrolled patients with vitiligo had NSV.

#### 3.1.2. Serum Concentrations of IFN-*γ*, IL-1*β*, and IL-6 among Patients with AA, NSV, and Healthy Controls

IFN-*γ*, IL-1*β*, and IL-6 serum levels were significantly elevated in AA patients and in NSV patients compared to healthy controls. Only IL-6 level was also significantly higher in NSV compared to AA patients (see Table 2). We found no significant sex difference in cytokines level of female and male patients with AA and NSV.

#### 3.1.3. Correlations between Serum Levels of IFN-*γ*, IL-1*β*, and IL-6 and Extent, Activity, and Duration of the Disease

The correlation between increased IL-6 serum levels and duration of the AA was confirmed in the Spearman test (*q* = 0.453; *p* = 0.010474). In AA patients, there were no correlations found between serum cytokine levels and extent (SALT)/activity of disease (VIDA) (see Table 3).

In NSV patients, the correlations between increased IL-1*β* serum levels and extent of vitiligo (VASI) (*q* = 0.383; *p* = 0.040441), duration of disease (*q* = 0.458; *p* = 0.012573) were revealed in the Spearman test. IL-1*β* serum level was negatively correlated with the activity of disease (VIDA) in NSV patients (*q* = −0.435; *p* = 0.018387), which may indicate that IL-1*β* in the initial stages of the disease has a local pathogenic effect on melanocytes (see Table 3).

## 4. Discussion

Oxidative stress and autoimmunity with genetic susceptibility have been associated with the pathogenesis of AA and vitiligo [2, 18–20]. The correlation between those two pathways is not fully understood. Heat-shock proteins, including Hsp60, Hsp70, and gp96, have been shown to exert cytokine-like effects on antigen-presenting cell (APC) maturation. These activities include the ability to enhance tumor necrosis factor-*α* (TNF-*α*), IL-1*β*, IL-6, and IL-12 secretion from monocytes, macrophages, and dendritic cells (DCs), to enhance surface expression of B7 and major histocompatibility complex (MHC) class II on DCs, to stimulate maturation and migration of DCs to draining lymph nodes, and to induce chemokine secretion by macrophages and DCs [21]. Enhanced Hsp70 is upregulated by IFN-*γ* from perilesional cytotoxic T lymphocytes (CTLs). Additionally, Hsp70 enhances IFN-*γ* release from CTLs through a positive feedback mechanism. The loop amplifies the process and exacerbates the destruction of vitiligo melanocytes [22]. Human genetic studies and functional studies have identified pathways critical for AA development, implicating a role for CD8+ T cells and IFN-*γ* in mediating hair follicle (HF) damage [23]. The study by Jacquemin et al.⁠ revealed that Hsp70 potentiated DNA-induced IFN-*α* production by plasmacytoid dendritic cells (pDCs) and subsequently IFN-*α*-induced expression of s and CXCL10 by keratinocytes [24]. Damaged cells upregulate stress ligands and IFN production. IFNs induce CXCL10 secretion from keratinocytes, which then attracts CXCR3-positive T cells [25]. Besides, TNF-*α*, IL-1*β*, and IFN-*γ* can induce another cytotoxic effector molecule, inducible nitric oxide synthase (iNOS) [26]. The inflammatory nature of AA/vitiligo and significant psychological stress associated with those diseases may further increase the levels of oxidative parameters.

In this study, we showed that the levels of IFN-*γ*, IL-1*β*, and IL-6 were higher in sera of AA and NSV patients compared to healthy controls. This may imply the role of these cytokines in both disease development and biology. The study has demonstrated the correlations between increased IL-1*β* serum levels and the extent of vitiligo (VASI) and also the duration of disease in NSV patients. IL-1*β* serum level was negatively correlated with the activity of disease (VIDA). These data may indicate that IL-1*β* at the initial stages of development of vitiligo is expressed mainly in the tissue.

We also demonstrated significantly increased IL-6 levels in NSV patients compared to AA patients. As established in previous studies serum IL-6 level increases in chronic diseases depending on the disease severity and location [27]. In our study, IL-6 levels were also positively correlated with the duration of the AA. Singh et al. have revealed similar findings, but in patients with vitiligo in whom the duration of disease was more than 15 years [28].

The studies show that IFN-*γ* is the key player in xanthine oxidase- (XO-) mediated oxidative stress. The role of xanthine oxidase (XO) in oxidative stress and its association with nitric oxide (NO)/NO synthase (NOS) have been widely reported [29]⁠. Proinflammatory IL-1*β* can also induce rapid expression of iNOS and generate a large amount of NO in tissues [30]⁠. Moreover, oxidative stress increases intercellular adhesion molecule-1 (ICAM-1) expression in epithelial cells through the IL-6/AKT/STAT3/NF-*κ*B-dependent pathway. Several studies indicate that the upregulation of ICAM-1 expression on epithelial cells is closely associated with proinflammatory cytokines, such as IL-6, IL-1*β*, and tumor necrosis factor-*α* (TNF-*α*)⁠ [31]. All those facts suggest an interesting link between oxidative stress and the serum cytokine profile in patients with AA and NSV. It indicates similar pathogenesis of both diseases.

As for the limitations of the study, we would like to mention that the number of subjects in the respective groups is quite small, but we employed suitable statistic methods for such groups. Furthermore, we did not measure direct stress markers such as superoxide dismutase, catalase, glutathione peroxidase, malondialdehyde, superoxide dismutase, catalase, hydrogen peroxide, nitric oxide, or total antioxidant capacity but could be the next step of our further studies in the future.

## 5. Conclusions

Our results supported the hypothesis that the elevated cytokines identified in this study could be the cause of oxidative stress observed in patents with AA and vitiligo, which can contribute to the onset of autoimmunity in genetically predisposed individuals. It suggests new perspectives in the advances in the understanding of both disease etiopathogeneses which involve interrelationships between oxidative stress and autoimmunity. Further studies exploring the effect of different oxidative stress markers and triggers in AA and vitiligo may be required to develop potential therapeutic strategies for those diseases. The JAK inhibitors that also suppress the response to oxidative stress have demonstrated promising results in promoting hair regrowth and repigmentation⁠.

## Figures and Tables

**Table 1:** Demographic and clinical characteristics of patients with alopecia areata (AA), patients with nonsegmental vitiligo (NSV), and healthy controls.

	AA patients	NSV patients	Controls
Total	33	30	30
Female/male, *n* (%)	21/12, 63.6/36.4	18/12, 60/40	23/7, 76.7/23.3
Average age ± SD (years)	18.64 ± 8.56	28.55 ± 19.23	19.95 ± 13.08
Female	18.76 ± 6.33	28.65 ± 19.58	18.40 ± 10.72
Male	18.44 ± 11.85	28.41 ± 19.54	15.05 ± 19.13
Mean SALT (AA)/VASI (NSV) ± SD	42.09% ± 33.81%	7.49 ± 8.28	n/a
Mean VIDA ± SD	3.06 ± 0.65	2.96 ± 0.33	n/a
Duration of disease ± SD	4.33 ± 5.075	7.63 ± 12.036	n/a
Age of onset	7.97 ± 8.17	14.59 ± 8.25	n/a

Data are expressed in *n*, percentage (%) or mean and standard deviation (SD). SALT: Severity of Alopecia Tool; VASI: Vitiligo Area Scoring Index; VIDA: Vitiligo Disease Activity Score; n/a: not applicable.

**Table 2:** Serum concentrations of IFN-*γ*, IL-1*β*, and IL-6 (pg/ml) among patients with alopecia areata (AA), patients with nonsegmental vitiligo (NSV), and healthy controls and a comparison of *p* values in the Mann–Whitney test among AA patients, NSV patients, and healthy controls. Data were considered to be statistically significant at a value of *p* < 0.05.

	AA patients (*n* = 33)	NSV patients (*n* = 30)	Controls (*n* = 30)	*p* value (AA patients vs. control)	*p* value (NSV patients vs. control)	*p* value (AA patients vs. NSV patients)
IFN-*γ*, mean±SD (pg/ml)	237.68 ± 55.05	226.10 ± 50.18	199.59 ± 41.27	*p* = 0.007283	*p* = 0.038467	*p* > 0.05
IL-1*β*, mean±SD (pg/ml)	300.09 ± 87.97	329.72 ± 85.24	219.61 ± 60.89	*p* ≤ 0.001	*p* ≤ 0.001	*p* > 0.05
IL-6, mean±SD (pg/ml)	121.38 ± 31.01	144.95 ± 33.46	75.26 ± 21.15	*p* ≤ 0.001	*p* ≤ 0.001	*p* = 0.004485

Data are expressed in *n*, mean and standard deviation (SD). IL: interleukin; IFN: interferon.

**Table 3:** The correlation between serum levels of interferon *γ* (IFN-*γ*), interleukin- (IL-) 1*β*, and IL-6 (pg/ml) and extent, activity, and duration of the disease among patients with alopecia areata (AA) and patients with nonsegmental vitiligo (NSV).

Correlation between serum cytokine levels and	Alopecia areata (AA)			Nonsegmental vitiligo (NSV)		
	Extent of AA (SALT)	Activity of AA (VIDA)	Duration of AA	Extent of NSV (VASI)	Activity of NSV (VIDA)	Duration of NSV
IFN-*γ* (q in the Spearman test/*p* value)	-0.055/0.759218	-0.206/0.250208	-0.150/0.421353	-0.055/0.778851	-0.148/0.444011	-0.137/0.478715
IL-1*β* (*q* in the Spearman test/*p* value)	0.038/0.833562	0.181/0.314325	-0.073/0.694889	0.383/0.040441	-0.435/0.018387	0.458/0.012573
IL-6 (*q* in trhe Spearman test/*p* value)	0.116/0.520565	-0.050/0.784260	0.453/0.010474	-0.054/0.779845	-0,190/0.323085	0,222/0,247687

SALT: Severity of Alopecia Tool score; VIDA: Vitiligo Disease Activity Score; VASI: Vitiligo Area Severity Index; IL: interleukin; IFN: interferon.

## Data Availability

The data used to support the findings of this study are available from the corresponding author upon request.

## References

[1] Deng Q., Wei J., Zou P. (2019). Transcriptome analysis and emerging driver identification of CD8+ T cells in patients with vitiligo. *Oxidative Medicine and Cellular Longevity*.

[2] Simakou T., Butcher J. P., Reid S., Henriquez F. L. (2019). Alopecia areata: A multifactorial autoimmune condition. *Journal of Autoimmunity*.

[3] Speeckaert R., van Geel N. (2017). Vitiligo: an update on pathophysiology and treatment options. *American Journal of Clinical Dermatology*.

[4] Boza J. C., Giongo N., MacHado P., Horn R., Fabbrin A., Cestari T. (2016). Quality of Life Impairment in Children and Adults with Vitiligo: A Cross-Sectional Study Based on Dermatology-Specific and Disease-Specific Quality of Life Instruments. *Dermatology*.

[5] Liu L. Y., King B. A., Craiglow B. G. (2016). Health-related quality of life (HRQoL) among patients with alopecia areata (AA): a systematic review. *Journal of the American Academy of Dermatology*.

[6] Rajabi F., Drake L. A., Senna M. M., Rezaei N. (2018). Alopecia areata: a review of disease pathogenesis. *British Journal of Dermatology*.

[7] Trüeb R. M., Dias M. F. R. G. (2018). Alopecia areata: a comprehensive review of pathogenesis and management. *Clinical Reviews in Allergy and Immunology*.

[8] Alikhan A., Felsten L. M., Daly M., Petronic-Rosic V. (2011). Vitiligo: a comprehensive overview. *Journal of the American Academy of Dermatology*.

[9] Ezzedine K., Lim H. W., Suzuki T. (2012). Revised classification/nomenclature of vitiligo and related issues: the Vitiligo Global Issues Consensus Conference. *Pigment Cell & Melanoma Research*.

[10] Taïeb A., Picardo M. (2009). Vitiligo. *The New England Journal of Medicine*.

[11] Dardalhon V., Korn T., Kuchroo V. K., Anderson A. C. (2008). Role of Th1 and Th17 cells in organ-specific autoimmunity. *Journal of Autoimmunity*.

[12] Wang C. Q. F., Cruz-Inigo A. E., Fuentes-Duculan J. (2011). Th17 cells and activated dendritic cells are increased in vitiligo lesions. *PLoS One*.

[13] Zhou L., Ivanov I. I., Spolski R. (2007). IL-6 programs T_H_-17 cell differentiation by promoting sequential engagement of the IL-21 and IL-23 pathways. *Nature Immunology*.

[14] Jabri B., Abadie V. (2015). IL-15 functions as a danger signal to regulate tissue-resident T cells and tissue destruction. *Nature Reviews Immunology*.

[15] Olsen E. A., Hordinsky M. K., Price V. H. (2004). Alopecia areata investigational assessment guidelines–Part II. *Journal of the American Academy of Dermatology*.

[16] Hamzavi I., Jain H., McLean D., Shapiro J., Zeng H., Lui H. (2004). Parametric modeling of narrowband UV-B phototherapy for vitiligo using a novel quantitative tool: the Vitiligo Area Scoring Index. *Archives of Dermatology*.

[17] Njoo M. D., Das P. K., Bos J. D., Westerhof W. (1999). Association of the Köbner phenomenon with disease activity and therapeutic responsiveness in vitiligo vulgaris. *Archives of Dermatology*.

[18] Wang Y., Li S., Li C. (2019). Perspectives of new advances in the pathogenesis of vitiligo: from oxidative stress to autoimmunity. *Medical Science Monitor*.

[19] Jimbow K., Chen H., Park J. S., Thomas P. D. (2001). Increased sensitivity of melanocytes to oxidative stress and abnormal expression of tyrosinase-related protein in vitiligo. *The British Journal of Dermatology*.

[20] Al-Shobaili H. A., Rasheed Z. (2015). Oxidized tyrosinase: a possible antigenic stimulus for non-segmental vitiligo autoantibodies. *Journal of Dermatological Science*.

[21] Millar D. G., Garza K. M., Odermatt B. (2003). Hsp70 promotes antigen-presenting cell function and converts T-cell tolerance to autoimmunity *in vivo*. *Nature Medicine*.

[22] Xie H., Zhou F., Liu L. (2016). Vitiligo: how do oxidative stress-induced autoantigens trigger autoimmunity?. *Journal of Dermatological Science*.

[23] Harris J. E. (2013). Vitiligo and alopecia areata: apples and oranges?. *Experimental Dermatology*.

[24] Jacquemin C., Rambert J., Guillet S. (2017). Heat shock protein 70 potentiates interferon alpha production by plasmacytoid dendritic cells: relevance for cutaneous lupus and vitiligo pathogenesis. *The British Journal of Dermatology*.

[25] Raam L., Kaleviste E., Šunina M. (2018). Lymphoid stress surveillance response contributes to vitiligo pathogenesis. *Frontiers in Immunology*.

[26] Ramirez-Ramirez V., Macias-Islas M. A., Ortiz G. G. (2013). Efficacy of fish oil on serum of TNF *α*, IL-1 *β*, and IL-6 oxidative stress markers in multiple sclerosis treated with interferon beta-1b. *Oxidative Medicine and Cellular Longevity*.

[27] Narazaki M., Kishimoto T. (2018). The two-faced cytokine IL-6 in host defense and diseases. *International journal of molecular sciences*.

[28] Singh S., Singh U., Pandey S. S. (2012). Serum concentration of IL-6, IL-2, TNF-*α*, and IFN*γ* in Vitiligo patients. *Indian Journal of Dermatology*.

[29] Singh D., Kumar V., Singh C. (2017). IFN-*γ* regulates xanthine oxidase-mediated iNOS-independent oxidative stress in maneb- and paraquat-treated rat polymorphonuclear leukocytes. *Molecular and Cellular Biochemistry*.

[30] Gu R., Shi Y., Huang W. (2020). Theobromine mitigates IL-1*β*-induced oxidative stress, inflammatory response, and degradation of type II collagen in human chondrocytes. *International Immunopharmacology*.

[31] Liu C. W., Lee T. L., Chen Y. C. (2018). PM2.5-induced oxidative stress increases intercellular adhesion molecule-1 expression in lung epithelial cells through the IL-6/AKT/STAT3/NF-*Κ*B-dependent pathway. *Particle and Fibre Toxicology*.

